# Changes in cesarean section rates after introduction of a punitive financial policy in Georgia: A population-based registry study 2017–2019

**DOI:** 10.1371/journal.pone.0271491

**Published:** 2022-07-19

**Authors:** Ingvild Hersoug Nedberg, Tinatin Manjavidze, Charlotta Rylander, Ellen Blix, Finn Egil Skjeldestad, Erik Eik Anda

**Affiliations:** 1 Faculty of Health Sciences, Department of Community Medicine, UiT The Arctic University of Norway, Tromsø, Norway; 2 National Center for Disease Control and Public Health, Tbilisi, Georgia; 3 Faculty of Health Sciences, Department of Nursing and Health Promotion, OsloMet–Oslo Metropolitan University, Oslo, Norway; ICMR National Institute of Medical Statistics (ICMR-NIMS), INDIA

## Abstract

**Background:**

There is little research on how financial incentives and penalties impact national cesarean section rates. In January 2018, Georgia introduced a national cesarean section reduction policy, which imposes a financial penalty on hospitals that do not meet their reduction targets. The aim of this study was to assess the impact of this policy on cesarean section rates, subgroups of women, and selected perinatal outcomes.

**Methods:**

We included women who gave birth from 2017 to 2019 registered in the Georgian Birth Registry (n = 150 534, nearly 100% of all births in the country during this time). We then divided the time period into pre-policy (January 1, 2017, to December 31, 2017) and post-policy (January 1, 2018, to December 31, 2019). An interrupted time series analysis was used to compare the cesarean section rates (both overall and stratified by parity), neonatal intensive care unit transfer rates, and perinatal mortality rates in the two time periods. Descriptive statistics were used to assess differences in maternal socio-demographic characteristics.

**Results:**

The mean cesarean section rate in Georgia decreased from 44.7% in the pre-policy period to 40.8% in the post-policy period, mainly among primiparous women. The largest decrease in cesarean section births was found among women <25 years of age and those with higher education. There were no significant differences in the neonatal intensive care unit transfer rate or the perinatal mortality rate between vaginal and cesarean section births in the post-policy period.

**Conclusion:**

The cesarean section rate in Georgia decreased during the 2-year post-policy period. The reduction mainly took place among primiparous women. The policy had no impact on the neonatal intensive care unit transfer rate or the perinatal mortality rate. The impact of the national cesarean section reduction policy on other outcomes is not known.

## Introduction

In an attempt to explain the observed rapid increase in cesarean section [CS] rates worldwide in the last decades, studies have found that both clinical, cultural, psychosocial, organizational and financial factors play a role [1]. The World Health Organization [WHO] does not recommend national CS rates higher than 10% because it does not contribute to reductions in maternal and newborn mortality [2]. Several countries currently have rates exceeding 50% [1]. The complexity contributing to the high CS rates means that trying to reverse an already high rate is difficult. Few have succeeded and those that did were at facility or regional levels [3–5].

WHO has recognized financial incentives (i.e., the added revenue hospitals can make from CS births) as a major driver of increasing CS rates [6] and attempts have been made to implement financial interventions to reduce CS rates (Taiwan and Brazil), but they failed to achieve a reduction [7,8]. A scoping review from 2020 concluded that the evidence on whether financial and regulatory strategies reduce CS rates is in inconclusive, due to the low quality and heterogeneity of the included studies [9]. Specifically, research is lacking on the impact of setting goals for CS rates at facility, regional, or national level, and how such goals affect maternal and neonatal outcomes [6].

After gaining independence from the Soviet Union in 1991, the republic of Georgia privatized their health system, a process complete by 2012. As a result, 80% of medical expenses were paid by the patients themselves [10]. To compensate for the high out-of-pocket payment, a package of state-funded health care covering 90% of the population was introduced in 2013, whereby hospitals are reimbursed for basic and emergency health care at fixed rates [11]. Notably, the ratio of reimbursement for a CS birth was set at 1.6 compared to a vaginal birth.

Georgian guidelines for labour and delivery do not recommend performing CS without a medical indication, yet the national CS rate has increased from 9% in 2000 to 44.7% in 2017 [12,13]. National health authorities have acknowledged the high CS rate as a problem, and in 2013, they set a goal to reduce the overall proportion of CS to 31% by 2020 and 27% by 2030 [14]. In January 2018, the Ministry of Internally Displaced Persons from the Occupied Territories, Health, Labour, and Social Affairs of Georgia introduced a national CS reduction policy, with set target rates for each hospital based on the CS rate from the previous year^.^ Hospitals are evaluated annually, and those not meeting their reduction targets must pay a financial penalty. To our knowledge, the Georgian approach has not been tried anywhere else in the world. The aim of this study was to assess the impact of the Georgian national CS reduction policy on CS rates, subgroups of women, and selected perinatal outcomes.

## Materials and methods

The Georgian Birth Registry (GBR) is a national, digital birth registry that was implemented on January 1, 2016, and made mandatory by law the same year. The GBR contains information from antenatal care visits (ANC), hospitalisations during pregnancy, labour, delivery, and the postpartum stay for both mothers and newborns. Details on the implementation of the GBR have been reported previously [13]. Each birth registered in the GBR is validated through the Vital Registration System, administrated by the National Center for Disease Control and Public Health and the Ministry of Justice. This study is reported as per the Strengthening the Reporting of Observational Studies in Epidemiology (STROBE) guidelines (S1 Checklist) [15].

### Setting

The perinatal regionalisation reform was initiated by the Ministry of Internally Displaced Persons from the Occupied Territories, Health, Labour, and Social Affairs of Georgia in 2015. The reform separated birth facilities into three levels: level 1 hospitals care for low-risk women and can perform emergency CS if necessary, level 2 hospitals constitute most facilities and care for both low-risk women and women with certain risk factors, while level 3 hospitals care for patients in need of intensive care and have access to blood banks in addition to other specialist services. The aim of the reform is to create a geographically structured system to select women to the appropriate level of care, securing that each region could provide tertiary care [11].

The national CS reduction policy was introduced on January 1, 2018, as part of the perinatal regionalisation reform. It directs hospitals to reduce their CS rate by 1% point if CS births were 31–35% the previous year, 3% points if CS births were 36–40%, 5% points if CS births were 41–45%, 7% points if CS births were 46–50%, 9% points if CS births were 51–55%, 12% points if CS births were 56–60% and 15% points if CS births were >60% the previous year. No clinical providers, hospital directors nor the media engaged in the lead up to the implementation of the policy. Although the policy is directed primarily toward Level 2 hospitals, it is a national policy; thus, we included hospitals of all levels in our analysis.

### Study design and study population

This population-based registry study includes all women registered in the GBR who gave birth between January 1, 2017, and December 31, 2019, at gestational age (GA)>22+0 weeks. Births with missing information on parity were excluded (n = 38), resulting in an analytical sample of 150 534 women, of whom 52 601 gave birth during the pre-policy period (January 1, 2017, to December 31, 2017) and 97 933 during the post-policy period (January 1, 2018, to December 31, 2019).

### Variables

The intervention of interest was the introduction of the national CS reduction policy on January 1, 2018. The main outcome of the study is overall CS rate. In addition, we hypothesized that rates of neonatal intensive care unit (NICU) transfer and perinatal mortality (PM) could be affected by the policy. These outcomes have been validated by merging data from the hospitalisation registry and the Vital Registration System and have been shown to be complete.

We extracted data from the GBR on delivery outcome (spontaneous vaginal, operative vaginal delivery (vacuum or forceps), CS) and recoded them as a binary variable (CS yes/no). Data on NICU transfers were obtained from the GBR and recorded as a binary variable. For PM, data on stillbirths (fetal death at GA ≥22 weeks or birthweight >500 g if GA is unknown) [16] and early neonatal deaths were extracted from the GBR and the Vital Registration System, respectively. All explanatory variables were extracted from the GBR, including maternal age (13–19, 20–24, 25–29, 30–34, ≥35 years), parity (0, 1, 2, ≥3), maternal education (primary, secondary, technical, higher education, unknown), GA at birth (22–31, 32–36, 37–38, 39–40, 41–43 weeks), and fetal presentation (cephalic, non-cephalic, other). Induction of labour, operative vaginal delivery, previous CS, ANC attendance, and multiple births were extracted as binary variables.

### Statistical analysis

Descriptive statistics of maternal and newborn characteristics are presented as mean values with standard deviations (SDs) for continuous variables, and as frequencies and percentages for categorical variables. To study the impact of the national CS reduction policy on rates of CS, NICU transfers, and PM, we used interrupted time series analysis (ITSA) to calculate the baseline mean rate (i.e., rate in January 2017), monthly trends in the pre-policy period, change in rate in the month following the policy change (i.e., in January 2018), and monthly trends in the post-policy period. Dependent variables were monthly rates of CS, NICU transfers, and PM. For CS, single-group ITSA was performed for CS overall, in addition to a multi-group analysis of nulliparous versus multiparous women. Multi-group ITSA was used to compare NICU transfer rates and PM rates among CS births and vaginal births. For these analyses, newborns, not births, were used as the denominator.

The ITSA relies on ordinary least square regression. We applied the Newey-West model to handle auto-correlation, which we assessed by a Cumby-Huizinga test. The key assumptions for ITSA models are that, without the intervention, the pre-intervention trend will continue into the post-intervention period, and that any time-varying confounding factors change slowly over time and will therefore not interfere when assessing the impact of a single policy implemented at a particular time point. The results from the ITSA models are presented graphically, and the regression parameters have also been tabulated.

Statistical analyses were performed using Stata/SE version 16.0 (Stata Corporation, College Station, TX, USA) using the ITSA-package [17].

### Ethical considerations

The GBR prepared an anonymised data set for this study. The National Center for Disease Control and Public Health Institutional Review Board, Georgia, approved the study protocol (IRB # 2017–010 31.03.2017), and the Regional Committee for Medical and Health Research Ethics of Northern Norway (REC North) approved the use of data from the GBR (2017/404/REC North). Informed consent was not relevant for this study as registration in the GBR is mandatory by law.

## Results

**Fig 1:**
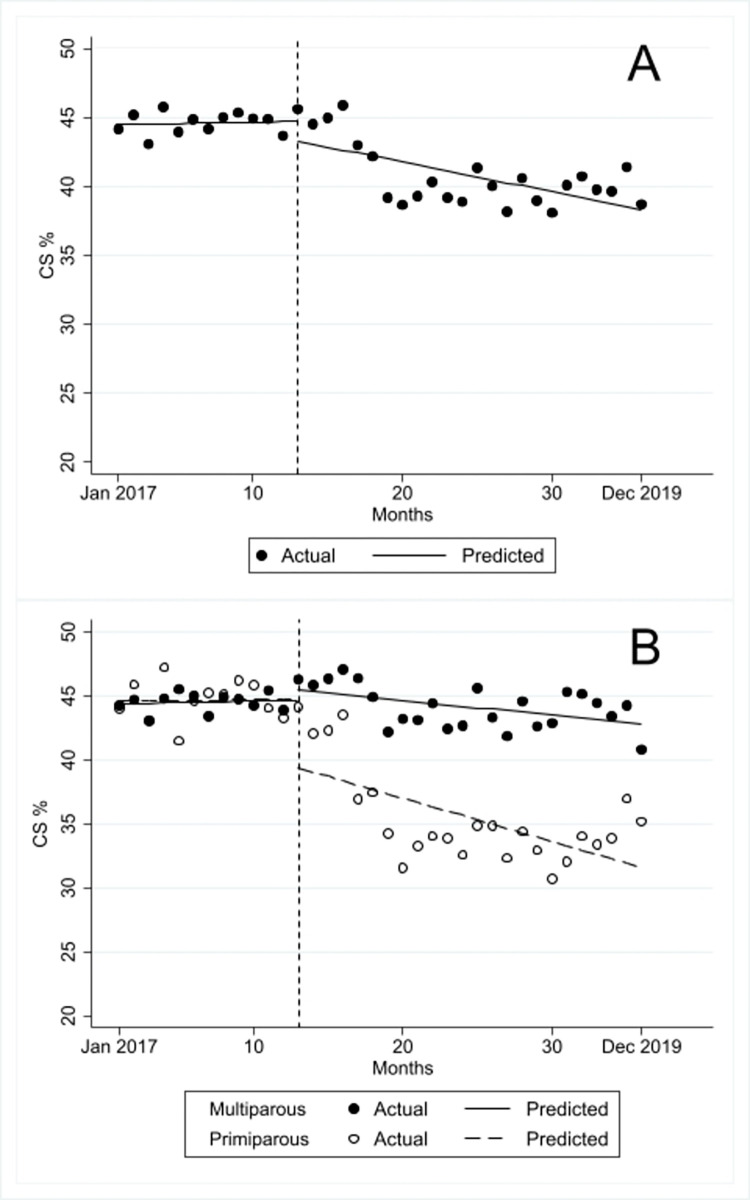
Cesarean section rates from January 2017 to December 2019 (pre-policy period: January-December 2017; post-policy period: January 2018-December 2019). A) Cesarean section rates overall B) Cesarean sections rates by parity.

The baseline mean CS rate in January 2017 was 44.47% and the trend was stable in the pre-policy period (Fig 1 & Table 1).

**Table 1:** Baseline rates, trends, and changes in cesarean section rates in the pre- (January-December 2017) and post-policy (January 2018-December 2019) period, including cumulative effect 2 years after policy change.

	Baseline mean cesarean section rate (i.e., in January 2017)	Monthly pre-policy trend	Change in the month following policy change (i.e., in January 2018)	Monthly post-policy trend	Change in post-policy relative to pre-policy trend	Cumulative effect 2 years after policy change
	%	% points per month (95% CI)	% points (95% CI)	% points (95% CI)	% points per month (95% CI)	% points
Overall/total	44.47	0.02 (-0.08 to 0.12)	-1.43 (-3.99 to 1.14)	-0.22 (-0.37 to -0.07)*	-0.24 (-0.40 to -0.07)*	-5.28
Primiparous	44.65	0.01 (-0.18 to 0.19)	-5.33 (-10.31 to -0.35)*	-0.34 (-0.64 to -0.04)*	-0.35 (-0.67 to -0.03)*	-8.16
Multiparous	44.40	0.03 (-0.05 to 0.10)	0.80 (-0.82 to 2.43)	-0.12 (-0.20 to -0.03)*	-0.14 (-0.27 to -0.02)*	-2.88

CI: Confidence interval.

* p-value <0.05.

In the month following the policy change, there was a statistically non-significant change in the CS rate of -1.43% points. Moreover, there was a reduction of 0.24% points in the monthly trend in the post-policy relative to the pre-policy period. The monthly trend decreased by 0.22% points in the post-policy period. The cumulative effect 2 years after the policy change was a total CS rate reduction of 5.28% points. When stratified by parity, the baseline mean CS rate was 44.65% for primiparous and 44.40% for multiparous women. The monthly trend was stable for both groups in the pre-policy period. In the month following the policy change, there was a sharp and statistically significant drop in the CS rate among primiparous women (-5.33% points, 95% confidence interval [CI]: -10.31 to -0.35, p = 0.036), while it remained stable for multiparous women. Relative to the monthly CS trend in the pre-policy period, a statistically significant decrease was observed in the post-policy period for both groups. The trend in the post-policy period showed a statistically significant decrease for both primiparous and multiparous women, but it was larger for primiparous women. The cumulative effect 2 years after the policy change was a reduction in the CS rate of 8.16% points among primiparous women and 2.88% points among multiparous women.

The mean CS rate in the pre-policy period was 44.6%, which decreased to 40.8% in the post-policy period (Table 2).

**Table 2:** Demographic presentation of the study population in the pre- (January-December 2017) and post-policy (January 2018-December 2019) periods. Cesarean sections in % of total number of births in each category.

	Pre-policy				Post-policy			
	CS (row %)		Total (column total %)		CS (row %)		Total (column total %)	
**Number of deliveries**	23 448	*44*.*6*	52 601		39 940	*40*.*8*	97 933	
**Maternal age, years**								
Mean (SD)	29.1 *(6*.*0*)		28.0 (*5*.*8*)		29.6 (*6*.*0*)		28.4 (*5*.*8*)	
13–19	1 166	*32*.*5*	3 590	*6*.*8*	1 576	*26*.*6*	5 917	*6*.*0*
20–24	5 182	*37*.*8*	13 703	*26*.*1*	8 063	*33*.*2*	24 276	*24*.*8*
25–29	7 279	*42*.*6*	17 099	*32*.*5*	12 333	*39*.*1*	31 515	*32*.*2*
30–34	5 599	*49*.*3*	11 347	*21*.*6*	10 076	*45*.*3*	22 243	*22*.*7*
≥2.	4 222	*61*.*5*	6 862	*13*.*1*	7 892	*56*.*4*	13 982	*14*.*3*
**Parity**								
0	9 413	*44*.*7*	21 061	*40*.*0*	13 086	*35*.*2*	37 137	*37*.*9*
1	9 149	*46*.*3*	19 775	*37*.*6*	16 418	*46*.*1*	35 630	*36*.*4*
2	3 813	*42*.*8*	8 918	*17*.*0*	7 819	*43*.*0*	18 186	*18*.*6*
≥8	1 073	*37*.*7*	2 847	*5*.*4*	2 617	*37*.*5*	6 980	*7*.*1*
**Maternal education** ^*^								
Primary	1 352	*31*.*3*	4 316	8.2	2 476	*30*.*5*	8 125	*8*.*3*
Secondary	9 687	*44*.*1*	21 992	41.8	15 475	*41*.*6*	37 245	*38*.*0*
Technical	1 458	*46*.*2*	3 154	6.0	2 393	*45*.*3*	5 280	*5*.*4*
Higher education	9 186	*47*.*9*	19 188	36.5	14 778	*42*.*4*	34 887	*35*.*6*
Unknown	1 763	*44*.*7*	3 948	7.5	4 818	*38*.*9*	12 396	*12*.*7*
**GA at birth, weeks**								
22–31	467	*53*.*2*	878	*1*.*7*	756	*49*.*3*	1 533	*1*.*6*
32–36	2 106	*64*.*9*	3 243	*6*.*2*	3 735	*58*.*5*	6 384	*6*.*5*
37–38	9 680	*59*.*0*	16 417	*31*.*2*	16 088	*52*.*4*	30 730	*31*.*4*
39–40	10 304	*36*.*0*	28 661	*54*.*5*	18 064	*33*.*9*	53 258	*54*.*4*
41–43	891	*26*.*2*	3 402	*6*.*5*	1 297	*21*.*5*	6 028	*6*.*2*
**Fetal presentation** ^**†**^								
Cephalic	18 404	*39*.*1*	47 108	*89*.*6*	31 964	*35*.*8*	89 393	*91*.*3*
Non-cephalic	3 428	*94*.*5*	3 627	*6*.*9*	6 354	*93*.*8*	6 774	*6*.*9*
Other	1 613	*86*.*6*	1 863	*3*.*5*	1 622	*91*.*9*	1 766	*1*.*8*
**Induction of labour**	83	*26*.*5*	313	*0*.*6*	424	*20*.*1*	2 111	*2*.*2*
**Operative vaginal delivery**			261	*0*.*5*			770	*0*.*8*
**Previous CS**	10 655	*99*.*9*	10 665	*20*.*3*	20 961	*99*.*9*	20 971	*21*.*4*
**ANC attendance**								
Yes	22 356	*45*.*0*	49 673	*94*.*4*	38 259	*41*.*2*	92 849	*94*.*8*
No	1 092	*37*.*3*	2 928	*5*.*6*	1 681	*33*.*1*	5 084	*5*.*2*
**Multiple births**	611	*83*.*0*	736	*1*.*4*	1 111	*77*.*9*	1 427	*1*.*5*

ANC: Antenatal care; CS: Cesarean section; GA: Gestational age; SD: Standard deviation.

* 3 missing in 2017.

^†^ 3 missing in 2017.

The CS rate decreased in all maternal age categories, but most notably in the youngest age groups (<25 years). For primiparous women, the mean CS rate decreased from 44.7% in the pre-policy period to 35.2% in the post-policy period, while there was minor change among multiparous women. There was also a decrease in the CS rate in all levels of maternal education, with the largest decrease taking place among women with higher education (from 47.9% to 42.4%). All categories of GA also displayed a decrease in CS rates, most notably in the early-term group (GA 37–38 weeks, from 59.0% to 52.4%) and in the post-term group (GA 41–43 weeks, from 26.2% to 21.5%). The CS rate among births with cephalic presentation decreased among those with cephalic presentation (39.1% to 35.8%). Induction of labour increased from 0.6% to 2.2%, while operative vaginal delivery increased from 0.5% to 0.8% in the post-policy period. The CS rate among women with a previous CS remained unchanged at 99.9%. CS rates decreased both for women who did and did not attend ANC visits, in addition to women with multiple births.

**Fig 2:**
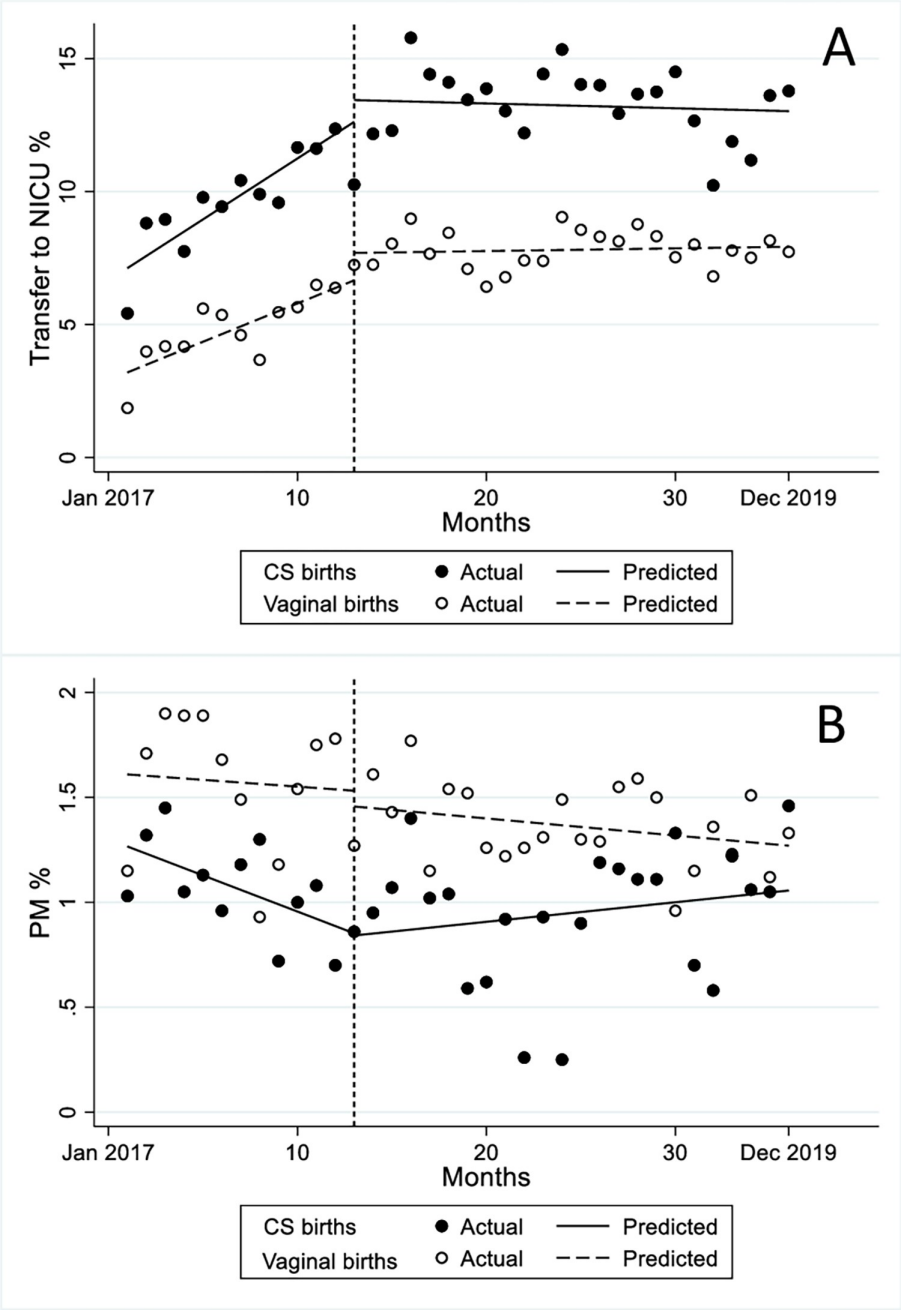
Perinatal outcomes from January 2017 to December 2019 (pre-policy period: January-December 2017; post-policy period: January 2018-December 2019). A) Transfer to NICU %, B) PM %. CS: Cesarean section; NICU: Neonatal intensive care unit; PM: Perinatal mortality.

The baseline mean NICU transfer rate was 7.12% for CS births and 3.20% for vaginal births, with a statistically significant increase for both types of birth in the pre-policy period (Fig 2 and Table 3).

**Table 3:** Baseline rates, trends, and changes in NICU transfer rates and perinatal mortality rates for cesarean section and vaginal births in the pre- (January-December 2017) and post-policy (January 2018-December 2019) period, including cumulative effect 2 years after policy change.

	Baseline mean rate (January 2017)	Monthly pre-policy trend	Change in the month following policy change (January 2018)	Monthly post-policy trend	Change in post-policy relative to pre- policy trend	Cumulative effect 2 years after policy change
	%	*% points per month (95% CI)*	*% points (95% CI)*	*% points*	*% points per month (95% CI)*	% *points*
**Transfer to NICU:** ***Cesarean births*** ***Vaginal births***	7.12 3.20	0.46 (0.35 to 0.57)* 0.29 (0.18 to 0.40)*	0.82 (-1.03 to 2.67) 1.03 (0.10 to 1.97)*	-0.02(-0.14 to -0.10) 0.01 (-0.03 to -0.05)	-0.48 (-0.63 to -0.32)* -0.28 (-0.39 to -0.17)*	-0.48 0.24
**Perinatal mortality:** ***Cesarean births*** ***Vaginal births***	1.27 1.61	-0.04 (-0.05 to -0.02)* -0.01 (-0.05 to 0.04)	-0.01 (-0.36 to 0.34) -0.08 (-0.40 to 0.25)	0.01 -(0.01 to 0.03) -0.01 (-0.01 to 0.003)*	0.04 (0.02 to 0.06)* -0.002 (-0.05 to -0.05)	0.24 -0.24

NICU: Neonatal intensive care unit.

* p-value<0.05.

In the month following the policy change, there was a significant increase in the NICU transfer rate among vaginal births (1.03% points, 95% CI: 0.10 to 1.97, p = 0.031), but not for CS births. Although both CS births and vaginal births showed a statistically significant decreasing trend in the post-policy versus the pre-policy period, the reduction was higher in the CS group (-0.48% points, 95% CI: -0.63 to -0.32, p<0.001). The observed increase in the monthly trend in the pre-policy period flattened in the post-policy period for both types of birth, with no significant difference between the two groups (-0.03% points, 95% CI: -0.16 to 0.10, p = 0.66) (not shown in table). Two years after the policy change, the cumulative effect on the NICU transfer rate was a decrease of 0.48% points for CS births and an increase of 0.24% points for vaginal births.

The baseline mean PM rate was 1.27% for CS births and 1.61% for vaginal births. There was a statistically significant decrease in the monthly trend in the pre-policy period for CS births (-0.04% points, 95% CI: -0.05 to -0.02, p<0.001). There was no statistically significant change in PM rates for either CS births or vaginal births in the month following the policy change. The change in the post-policy versus the pre-policy trend was statistically significant for CS births (0.04% points, 95% CI: 0.02 to 0.06, p<0.001). There was no statistically significant difference between the two groups in the post-policy period (0.02% points, 95% CI: -0.001 to 0.04, p = 0.06, not shown in the table). Two years after the policy change, there was a cumulative increase in the PM rate of 0.24% points among CS births and a cumulative decrease of 0.24% points among vaginal births.

## Discussion

The 2018 national CS reduction policy led to a reduction in the national CS rate from 44.7% in the pre-policy group to 40.8% in the post-policy group. The largest decrease was observed among primiparous women across all age groups. The decrease was also notable in women with higher education and in early- and post-term births. There was no significant difference in rates of NICU or PM between vaginal and CS births in the post-policy period.

Existing literature on financial interventions to reduce high CS rates is characterized by large variations in interventions, duration of assessment and methodology, making comparisons difficult [9,18]. To our knowledge, this is one of the few studies that assess a national CS reduction policy using population-based data in a middle-income country. Georgia chose to implement a single policy to reduce CS births, where no maternal health providers engaged in its development or implementation. Such a strategy is not recommended due to the substantial risk of failure by not considering the complexity of factors contributing to high CS rates [18]. Taiwan is an example, where raising, and thereby equalizing the reimbursement of vaginal births and CS births, yielded little or no results in reducing the national CS rate [8,19]. Our findings are in direct contrast with these results, but one could argue that, with the high baseline CS rate of 44.7% in Georgia, the reduction in CS births observed two years after the policy change could be classified as “low-hanging fruit”. Another difference is that Taiwan, together with other countries which has introduced financial measures, employed an incentive-based approach by increasing reimbursement for vaginal births [8,19–22]. Georgia instead implemented a punitive measure, where hospitals both are reimbursed less since they must perform fewer CS, and they are fined if they do not meet the set reduction targets. This type of financial penalty can have unintended consequences, such as clinicians performing more complicated vaginal deliveries that should be managed by CS. Our findings did not find a significant difference between vaginal and CS birth for NICU transfer and PM rates in the post-policy period, but this does not mean the policy has not impacted other outcomes not assessed in this study. Unfortunately, there are no other studies on financial or regulatory strategies to reduce CS rates that included perinatal outcomes. The generalizability of our results should be applied with caution, but financial interventions could represent a reduction strategy in countries with high CS rates and a similar reimbursement system, although the long-term implications of such a policy are unknown.

Studies from regions of Brazil and China, two countries with some of the highest CS rates in the world, show examples of successful, multi-intervention strategies that reduced high CS rates [5,23]. These interventions included both maternal health education on the benefits of vaginal delivery, public campaigns, introduction of indicators of normal birth, education of health care providers, training in complicated vaginal deliveries, and creation of a culture that encourages natural childbirth. This kind of multi-faceted approach that aims to change obstetric culture and practice over time could be a useful avenue of exploration for Georgian authorities and other countries interested in reducing high CS rates, including scaling up the use of, promoting the role of, and affording more autonomy to midwives during pregnancy and childbirth [24,25]. Such approaches should also include a better understanding of pregnant women’s preferred mode of delivery and a focus on woman-centred care. The challenge for Georgia will therefore be to make the change sustainable and progressive, without compromising maternal and newborn health.

The observed reduction in CS rates in primiparous women is not surprising, since Georgia maintained a CS rate of 99.9% for women with a previous CS throughout the study period. This reduction among primiparous women will probably lead to a reduction in the CS rate among multiparous women in the following years, since the risk of CS after a first vaginal delivery is low. The largest decreases in CS rates were seen in the youngest age groups. This finding agrees with the largest reduction observed among primiparous women, but we did observe a decrease in the oldest age group as well. For maternal education, the largest decrease was seen among women with higher education. This finding resonates with other studies from low- and middle-income countries, which have shown that overuse of CS is strongly associated with high maternal education [26–29]. Attempts to reduce CS without a medical indication will therefore be apparent in this group. The proportion of CS in the non-cephalic group remained high and stable both in the pre- and post-policy periods. This is to be expected, since Georgia practices elective CS for breech presentation. Interestingly, the CS rate decreased in all GA groups, most notably in the post-term group and the early-term group. Compared to 17 European countries and the United States [30], the CS rates in Georgia for GA 32–36 and 37–38 weeks were much higher both in the pre- and post-policy periods, indicating that factors other than medical emergency are responsible for the observed high rate. There was a slight increase in the rates of operative vaginal delivery and induction of labour, but it is too early to say if this is an indication that clinicians are considering these procedures as an alternative to elective or emergency CS. Lack of experience and confidence in performing operational vaginal delivery among obstetricians have been found to be associated with an increased use of CS [31].

## Strengths and limitations

A strength of the study is the large and comprehensive study sample, and the use of data from a national birth registry with close to 100% coverage. Another strength is that the perinatal outcomes were validated. A weakness of the study is that we could not differentiate between elective and emergency CS in the study due to suspected misclassification of CS in the GBR [32]. CS performed without a medical indication is reimbursed as if it were a vaginal birth, meaning a loss of revenue for the hospital in question. Thus, there are no incentives to classify a CS as a non-medically indicated intervention. The ability to distinguish between medically indicated and non-medically indicated CS would have been important to evaluate the effect of the Georgian national CS reduction policy on maternal and newborn outcomes. Although the assessment of maternal outcomes would have provided a more comprehensive picture of the impact of the policy, we did not include them because these outcomes have not been properly validated. The outcome PM is relatively rare, and proportions presented are subject to random fluctuations. Subsequently, changes in these proportions (or even lack thereof) should be interpreted with caution. Another weakness is the short follow-up period after the introduction of the CS policy. On the other hand, if no change had been detected after 2 years, the policy would have been deemed a failure. Although a prerequisite of ITSA is that no other intervention is introduced at the same time as the intervention under study, Georgia changed the number of recommended ANC visits from four to eight in January 2018. However, we do not believe that this impacted our perinatal outcomes, as a previous study found that this increase in visits did not change the proportion of NICU admissions or PM [33].

## Conclusion

Georgia has managed to reduce their national CS rate following the introduction of a punitive financial policy. Our findings indicate that CS rate reduction strategies may not increase PM. The results of this single-intervention policy indicate that financial policies do have a role to play in reducing high CS rates internationally, but they need to be closely monitored to avoid possible unintended consequences that could affect maternal and newborn outcomes. Georgia should consider multi-intervention strategies to reduce their high CS rate, such as strengthening the role of midwives and incorporate the views of pregnant women.

## Supporting information

S1 Checklist STROBE checklist strengthening the reporting of observational studies in epidemiology. (DOCX)
